# Carbon Footprint Assessment of Spanish Dairy Cattle Farms: Effectiveness of Dietary and Farm Management Practices as a Mitigation Strategy

**DOI:** 10.3390/ani10112083

**Published:** 2020-11-10

**Authors:** Ridha Ibidhi, Sergio Calsamiglia

**Affiliations:** 1 Department of Eco-Friendly Livestock Science, Institute of Green Bio Science and Technology, Seoul National University, Pyeongchang, Gangwon 08826, Korea; ridha@snu.ac.kr or; 2 National Institute of Agronomic Recherche of Tunisia (INRAT), Laboratory of Animal Production and Forages, Université de Carthage, rue Hédi Karray, Ariana 2049, Tunisia; 3 Animal Nutrition and Welfare Service (SNiBA), Departament de Ciència Animal i dels Aliments, Universitat Autònoma de Barcelona, 08193 Bellaterra, Spain

**Keywords:** greenhouse gas, carbon footprint, dairy farm, methane

## Abstract

**Simple Summary:**

Livestock production has been identified as an important source of greenhouse gas emissions. The current study was conducted to quantify the carbon footprint of Spanish dairy farms and to evaluate the potential of nutritional and management practices for mitigating methane emissions at farm level. The carbon footprint ranged from 0.67 to 0.98 kg CO_2_-eq/kg of energy corrected milk. Simulation scenarios showed that methane emissions and the carbon footprint of milk could be reduced more through management practices rather than dietary strategies. Modelling may provide policy makers, farmers and stakeholders valuable information for planning and developing strategies to reduce the carbon footprint associated with milk production.

**Abstract:**

Greenhouse gas emissions and the carbon footprint (CF) were estimated in twelve Spanish dairy farms selected from three regions (Mediterranean, MED; Cantabric, CAN; and Central, CEN) using a partial life cycle assessment through the Integrated Farm System Model (IFSM). The functional unit was 1 kg of energy corrected milk (ECM). Methane emissions accounted for the largest contribution to the total greenhouse gas (GHG) emissions. The average CF (kg CO_2_-eq/kg of ECM) was 0.84, being the highest in MED (0.98), intermediate in CEN (0.84), and the lowest in CAN (0.67). Two extreme farms were selected for further simulations: one with the highest non-enteric methane (MED1), and another with the highest enteric methane (CAN2). Changes in management scenarios (increase milk production, change manure collection systems, change manure-type storage method, change bedding type and installation of an anaerobic digester) in MED1 were evaluated with the IFSM model. Changes in feeding strategies (reduce the forage: concentrate ratio, improve forage quality, use of ionophores) in CAN2 were evaluated with the Cornell Net Carbohydrate and Protein System model. Results indicate that changes in management (up to 27.5% reduction) were more efficient than changes in dietary practices (up to 3.5% reduction) in reducing the carbon footprint.

## 1. Introduction

Climate change is a worldwide concern and the carbon footprint (CF) was proposed as an indicator to tackle the greenhouse gas (GHG) emissions from anthropogenic activities to the atmosphere [1]. Agricultural production, in general, and livestock in particular is recognized as an important contributor to GHG production [2]. The global livestock population contributes about 14.5% to global GHG emissions and the dairy sector is estimated to contribute by 4% of all anthropogenic GHG emissions [3,4]. Driven by population growth and diet changes towards an increase meat and dairy consumption, the production of livestock products globally is increasing. This increase will carry significant environmental costs through the accumulation of GHG in the atmosphere [5]. 

The main sources and types of GHG from ruminants include methane (CH_4_) production from enteric fermentation and animal manure, carbon dioxide (CO_2_) from land use, and nitrous oxide (N_2_O) from manure and slurry management [6]. Mathematical modelling approaches, including life cycle assessment (LCA), Intergovernmental Panel on Climate Change (IPCC) guidelines [6,7], and predictive models (empirical and mechanistic), have been used to evaluate the contribution of livestock production to GHG emissions [8,9]. Many studies have been conducted to quantify GHG emissions from individual farm sources, but few have provided estimates of total farm-level emissions [10]. Therefore, whole farm predictive models were proposed to estimate the CF and the profile of GHG emissions by production stage integrating all sources of emissions [9,11]. In addition, whole farms models can be used as an effective tool to evaluate the overall impact of management strategies to reduce farm gate GHG emissions. The Integrated Farm System Model (IFSM) is a process-based mechanistic whole-farm simulation model that incorporates soil processes, crop growth, tillage, planting and harvest operations, feed storage, feeding, herd production, manure storage, and economics [12]. The IFSM was originally used in dairy farms in the United States [13] and successfully adapted to be used in dairy farms in Australia and Canada [14,15]. 

The Spanish dairy sector is a major industry with about 850,000 milking cows producing 7,100,000 ton/year of raw milk [16]. Furthermore, Spain is committed to the United Nations Framework Convention on Climate Change (UNFCCC) and annually reports its national emissions inventory following guidelines promoted by the IPCC [6] to limit or reduce GHG from different sectors through national measures. Previous studies conducted in Spain used different methods to estimate the CF of milk from dairy cattle [17,18]. Del Prado et al. [17] assessed the CF of milk from 17 commercial confined dairy farms in the Basque Country (northern Spain) using a combination of LCA [19], NGAUGE [20], and LAND_DAIRY_ [13,21] models. Results revealed that the CF of milk ranged from 0.84 to 2.07 kg CO_2_-eq/kg energy corrected milk (ECM). Laca et al. [18] also reported that the milk CF (CO_2_-eq/kg fat-protein corrected milk) was higher in the semi-confined system (1.22 kg) compared with the pasture-based system (0.99 kg) in northern Spain. Despite the significant contributions of these studies, none has yet compared results from different regions and assess the impact of feeding and management changes on CH_4_ emissions and the milk CF. 

The objectives of the current study were: (1) to predict GHG emissions and assess the CF from twelve dairy farms from the three most productive regions in Spain; (2) to evaluate the diet contribution on enteric CH_4_ emissions; and (3) to evaluate the impact of dietary and management modifications on CH_4_ emissions and the milk CF.

## 2. Materials and Methods 

### 2.1. Farm Selection and Data Collection

This study was carried out in the three most important regions in dairy production in Spain and with the highest potential for future expansion: Mediterranean (MED: Catalonia, Valencia and Murcia), Cantabric (CAN: Galicia, Asturias and Cantabria) and Central (CEN: Castilla-La Mancha, Castilla-Leon, Madrid and Aragon). MED has predominantly a Mediterranean climate, CAN has an Atlantic climate and CEN has a temperate continental climate [22]. CAN is home of 52% of the total dairy cattle milk production, while MED and CEN represent 14 and 20% of the total milk production, respectively [16]. 

Farm data were collected through a questionnaire from twelve Holstein dairy farms in Spain, four from each region. Selected dairy farm represented a significant number of dairy farms in a region in terms of size, forage and crops grown, livestock systems, labor organization, management practices and production technology used [23]. Within each region, farms were selected in a two-step process: In the first step, technical advisors with a sound knowledge of local conditions and with good contacts with farm managers were contacted. The main characteristics of farms were discussed with the aim to describe a typical farm for each region. In the second step, selected farms data were obtained by visiting the farms and completing a structured questionnaire. The questionnaire collected information from different areas: crop and soil, grazing, machinery, tillage and planting, crop harvest, feed storage, facilities, bedding type, herd productivity, feeding strategies and manure management. Diet composition from each cattle group of each herd was also requested (type and amounts of forages and concentrates) together with nutritional management strategies (feeding groups for each herd, feeding system, grazing activity and mineral supplementation). The general characteristics of the selected dairy farms from each region are presented in Table 1. 

### 2.2. Modeling Procedure

**Figure 1:**
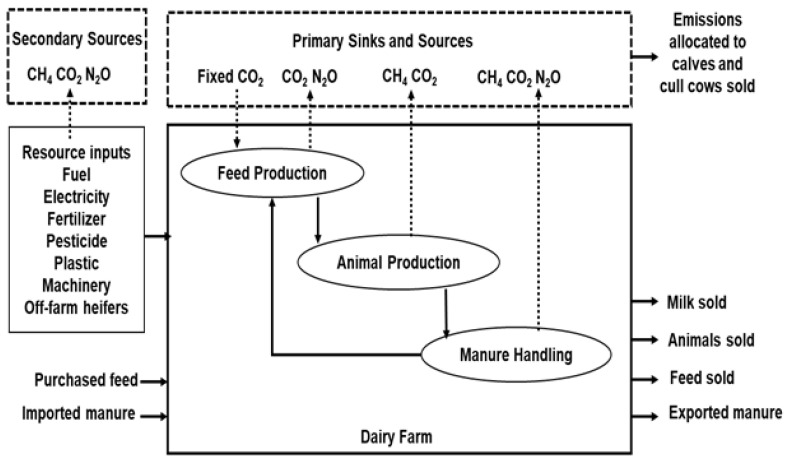
System boundaries, primary and secondary greenhouse gas emissions sources and sinks for a partial life cycle assessment of the carbon footprint (adopted from Rotz et al. [13]).

Farm emissions were simulated using the IFSM, a mechanistic model developed and validated by Chianese et al. [12] to calculate CH_4_, N_2_O, CO_2_ and total GHG emissions from dairy cattle production systems through a partial LCA. The IFSM was further refined into a process-based whole farm simulation including major components for soil processes, crop growth, tillage, planting, harvesting, feed storage, feeding, herd production, manure storage and economics in dairy farms (Figure 1) [24]. The functional unit considered in this study was 1 kg of ECM at the farm gate. The system boundaries contained all relevant activities from cradle to farm gate including on-farm activities, the production of farm inputs and carbon sequestration. Emissions attributable to imported feeds and its transportation were excluded. In addition, the model was adapted to be used in Spanish conditions such as soil type, nutritional characteristics of forages and concentrates, and the 10-year average daily local weather. Weather variables included annual temperature, relative humidity, wind velocity, mean precipitation and solar radiation from three meteorological stations corresponding to each region.

In addition, the Cornell Net Carbohydrate and Protein System Model (CNCPS version 6.1; [25]) was used to evaluate the contribution of diets to enteric CH_4_ emissions of lactating cows on each farm and explore the impact of dietary modifications on CH_4_ emissions. The CNCPS is a mechanistic mathematical model that estimates cattle requirements and nutrient supply based on animal, environment, and feed composition information and predicts nitrogen (N), phosphorus (P) and CH_4_ emissions, enabling its integration in a whole-farm nutrient management plans [25,26].

### 2.3. Methane Mitigation Scenarios Simulation

To assess how changing dietary and management practices affects the enteric, non-enteric CH_4_ emissions and milk CF, different scenarios were compared. Two extreme dairy farms were selected for further simulations: one with the largest enteric CH_4_ emissions, and another with the largest non-enteric CH_4_ emissions.

The CNCPS model [25] was used in the farm with the largest share of enteric CH_4_ emissions to evaluate three changes in the diet composition of milking cows to determine how these changes would affect enteric CH_4_ emissions:

(i) Reducing by 10% the forage to concentrate ratio;
(ii) Improving forages quality: the original ryegrass silage (9% crude protein, CP; 65% neural detergent fiber, NDF; and 8% lignin, ADL) was changed by an improved ryegrass silage (21% CP, 50% NDF and 7% ADL);
(iii) Supplying 330 mg/day of monensin.

The IFSM model was used in the farm with the largest share of non-enteric CH_4_ emissions to analyze the impact of five management changes on CH_4_ emissions:

(i) Changing milk production from 9565 to 11,000 (+13%) and 8000 (−15%) kg milk/cow/year;
(ii) Comparing five manure collection systems: top-loaded lined earthen basin, top-loaded lined concrete tank, bottom-loaded tank, covered tank and enclosed tank;
(iii) Comparing four types of manure storage: semi-solid (12–14% dry matter; DM), solid (20% DM), slurry (8–10% DM) and liquid-slurry (5–7% DM);
(iv) Comparing five types of bedding: straw, chopped straw, sand, sawdust and manure solids.
(v) Installing an anaerobic digester.

## 3. Results

### 3.1. Greenhouse Gas Emissions Profile and the Carbon Footprint of the Selected Dairy Farms

The estimated annual averages for CH_4_, N_2_O, CO_2_, total GHG emissions and CF of milk (kg of CO_2_-eq/kg of ECM) from dairy cattle farms by source of emissions (animal housing, manure storage, manure field application, grazing, fuel combustion and secondary emissions) from MED, CAN and CEN regions are presented in Table 2. The annual average CH_4_ emissions were the highest in MED (328 ± 25.5 kg/cow), intermediate in CEN (273 ± 52.0 kg/cow) and the lowest in CAN (251 ± 48.0 kg/cow). The highest annual average N_2_O emissions were in MED (6.5 ± 1.81 kg/cow), intermediate in CEN (4.0 ± 1.52 kg/cow) and the lowest in CAN (3.2 ± 1.66 kg/cow). Negative values were obtained for the annual average net CO_2_ emissions on all farms as the positive contributions from animal and housing, manure storage, grazing and fuel combustion were offset by the net feed production. The average CF of milk in selected farms was 0.87 kg CO_2_-eq/kg of ECM, being the highest in MED (1.0 ± 0.12), intermediate in CEN (0.8 ± 0.08) and the lowest in CAN (0.7 ± 0.07 kg of CO_2_-eq/kg of ECM). The breakdown of total GHG emissions into component gases (enteric CH_4_, non-enteric CH_4_, N_2_O and CO_2_) was examined in absolute terms and relative to each other for all farms. 

CH_4_ emissions from enteric fermentation and manure handling (non-enteric fermentation) represented the largest share of GHG emissions, accounting for more than 70% of total farm emissions.

### 3.2. Diet Evaluation with the CNCPS Model and Its Contribution to Enteric Methane Emissions

Diet evaluation of each farm with the CNCPS model and its contribution to enteric CH_4_ emissions per unit of milk produced are presented in Table 3. The highest enteric CH_4_ emissions (g/kg of milk) were recorded in CAN farms with an average of 13.6 and a range from 12.2 in the CAN1 to 16.2 in the CAN2 farms. Intermediate CH_4_ emissions (g/kg of milk) were recorded in MED with an average of 12.5 and a range from 11.2 to 13.3 in the MED3 and MED4 farms, respectively. The lowest CH_4_ emissions (g/kg of milk) were recorded in CEN farms with an average of 12.4 and a range from 10.7 to 14.4 in CEN4 and CEN1 farms, respectively.

### 3.3. Characteristics of the Two Selected Extreme Farms

Two extreme dairy farms in term of CH_4_ emissions were selected for further simulations (Table 4): one with the largest non-enteric CH_4_ emissions (MED1), and the other with the largest enteric CH_4_ emissions (CAN2). The first farm was used to simulate the effects of changes in management on non-enteric CH_4_ emissions using the IFSM model. Farm MED1 is located on a 330 ha clay loam soil in Girona that is used to provide most of the forage required for feeding cows (ryegrass and corn silage). It has 82 milking Holstein cows plus replacement heifers housed in a free stall barn and bedded pack barns, respectively. Average intake was 26.5 kg DM of a 55:45 forage to concentrate ratio, and average production was 32.0 L/cow/day. The second farm was used to simulate the effects of changes in diet composition on enteric CH_4_ emissions using the CNCPS model. The CAN2 farm is on a 25 ha of loam soil in Lugo (northern Spain) that produces all required ryegrass and forage for pasture. It has 42 milking Holstein cows housed in a free stall barn with access to pasture. Average estimated intake and production of milking cows was 21.3 kg DM (68:32 forage to concentrate ratio) and 28.5 L/cow/day of milk, respectively. 

### 3.4. Dietary and Management Changes Scenarios to Reduce Enteric and Non-Enteric Emissions

#### 3.4.1. Dietary Change Scenarios

The CNCPS model was used to analyze three potential changes in diet composition and quality in the CAN2 farm to determine how these changes affected their enteric CH_4_ emissions. Results of the simulated scenarios are shown in Table 5. 

The 10% reduction in the forage: concentrate ratio reduced CH_4_ emissions from 16.4 to 15.6 g/kg milk, representing a 5% reduction in CH_4_ emissions, but only 3.5% of the milk CF. In contrast, change in the nutritional value of the ryegrass silage had a small impact on CH_4_ emissions which decreased from 16.4 to 16.3 g/kg milk. Finally, the addition of 330 mg/day monensin also had small effects on CH_4_ emissions from 16.4 to 16.3 g/kg milk. The impact of these two dietary factors on the milk CF was negligible.

#### 3.4.2. Management Change Scenarios

The effects of management changes on CH_4_ emissions and the CF in the MED1 farm are presented in Table 6. Simulation with the IFSM revealed that the increased milk production by 13% reduced slightly CH_4_ emissions (−0.2%), but the impact on the milk CF was moderate (−6.4%). Change in bedding types from straw to sand had a larger effect, reducing CH_4_ emissions by 10% and the milk CF by 13.8%. Farm MED1 has already a semi-solid manure management system that it is rather efficient, reducing CH_4_ emissions around 32% and the milk CF by 7.4% compared with slurry or liquid-slurry systems. The utilization of a solid manure management system would further reduce the milk CF by 17.4%. The construction and use of an anaerobic digester would reduce CH_4_ emissions by 29.9% and the milk CF by 24.8%. The highest reduction potential would be obtained if the top-loaded lined earthen basin is changed into an enclosed tank, with a 37.5% and 27.5% reduction in CH_4_ emissions and the milk CF, respectively. 

## 4. Discussion

### 4.1. The Carbon Footprint

The milk CF among all farms ranged from 0.6 to 1.1 kg CO_2_-eq/kg ECM and was within the range reported in earlier LCA studies using the same model for conventional dairy farms [13,15]. Del Prado et al. [17] reported a slightly higher CF (0.84 to 2.07 kg CO_2_-eq/kg ECM) in 17 commercial confined dairy farms in the Basque Country (northern Spain) using a combination of sub-models approaches. Laca et al. [18] also reported a higher CF of milk in semi-confined dairy cows in northern Spain (1.22 kg CO_2_-eq/kg ECM). Differences compared with our results are explained by the different system boundaries delimitation used, where emissions from transport and purchased feeds were included in these previous studies but not in ours, which justifies higher values. In contrast, the milk CF in Pennsylvania and California farms were lower (0.60, 0.55 and 0.61 kg CO_2-_eq/kg ECM, respectively) [13] than results reported herein. Belflower et al. [27] found that the CF in conventional United States (USA) farms was about 0.87 kg CO_2_-eq/kg ECM, closer to our estimates, but variation was much larger in Holstein cows in Australia (range from 0.54 to 1.35 kg CO_2_-eq/kg ECM; [15]). Results obtained in our simulations are within the range of those reported elsewhere in similar conditions. 

### 4.2. Methane and Carbon Footprint Mitigation Strategies

CH_4_ is one the main GHG produced at farm gate from enteric and non-enteric fermentation in dairy farms which mitigation may help reduce the CF [28,29,30]. It is very important to identify which strategies may have the highest impact on the reduction of CH_4_ emissions and the milk CF, so policy-makers, farmers and stakeholders may direct their policies toward the most efficient processes. In the current study, we evaluated the effects of dietary and management changes on CH_4_ emissions and milk CF using CNCPS and IFSM, respectively.

#### 4.2.1. Dietary Change Scenarios

Dietary changes in the CAN2 farm were simulated with the CNCPS model, and reformulated to optimize production using the ingredients available in the farm. Our results suggest that CH_4_ emissions originated from enteric fermentation and the CF of milk could be reduced up to 5.0% and 3.5%, respectively, when the proportion of forage was reduced by 10%. Feeding grain increases ruminal propionate while lowering acetate levels from microbial fermentation [29,30,31]. While fibrolytic bacteria ferment toward acetate, which releases a carbon that methanogenic bacteria reduce with metabolic hydrogens to produce CH_4_, amylolytic bacteria ferment towards propionate that consumes metabolic hydrogens. Therefore, when the fermentation is shifted towards a lower acetate to propionate ratio, CH_4_ production is reduced and justifies the results observed. Also, the increase in concentrate in the diet resulted in an increase in milk production that contributed to the dilution of the amount of CH_4_ per kg of milk. In contrast, improving silage quality had negligible impact on reducing CH_4_ emissions and the milk CF. The small impact of an improved silage quality was expected. Most dairy diets in Spain contain under 55% forage in their diets, and quality is already reasonably high. Increasing forage quality implies a better digestibility of the fiber components, but probably has a small impact on the fermentation pathways, producing similar proportions of CH_4_. There are few in vivo data exploring the impact of forage quality on CH_4_ emissions and the milk CF. Boadi and Wittenberg [32] reported that CH_4_ emissions in dairy heifers were reduced by 23.4 and 42.5% when forage quality improved from low to medium and high quality silage, respectively. However, the high proportional impact is counterbalance with the small overall contribution of young non-producing animals to the overall farm GHG production. Finally, the impact of monensin on CH_4_ emissions observed in our report was small. Monensin decreased the acetate-to-propionate ratio in rumen fluid through increasing the flow of reducing equivalents to form propionate [33]. In fact, results from a meta-analysis conducted based on 11 experiments on lactating dairy cows showed that monensin supplementation reduced CH_4_ production by about 2−3% [34]. It is possible that the small changes reported herein result from an underestimation of the effects of monensin on CH_4_ emissions in the CNCPS model. However, the effects of monensin, even considering in vivo evaluations, are relatively small. All dietary changes proposed herein are within reasonable changes to be implemented in a dairy farm. Therefore, the overall potential of dietary changes appears to have a relatively small impact of up to 5% in the reduction of CH_4_ emissions and up to 3.5% in the CF. 

#### 4.2.2. Management Change Scenarios

On the other hand, the IFSM model was used to simulate management scenarios in the MED1 farm with the highest non-enteric CH_4_ emissions. Three major strategies should be considered: increase productivity to dilute fixed GHG emissions; use bedding material that produce less emissions; and modify manure management practices. 

Increasing productivity of milking cows is an effective strategy to mitigate GHG emissions, which may allow a reduction in animal numbers providing the same edible product output at a reduced CF. We estimated that an increase of around 1.500 L/cow/year would reduce the CF of milk by 6.4%. Boadi and Wittenberg [32] also reported that increasing milk production of dairy cows from 5000 to 10,000 L/cow/year in the European Union would increase total CH_4_ production per animal by 23%, but the production/ECM would be reduced up to 40%. In fact, the sustained increase in productivity was the single most influential mitigation factor which reduced the CF of milk in the United States dairy industry from 1944 to 2007 [35]. 

Bedding material may also have a relevant impact on CH_4_ emissions and the milk CF. The MED1 farm had a straw-type bedding. While differences in CF compared with other organic alternatives were small, the use of sand would have a moderate, yet relevant, impact, reducing CH_4_ emissions by 9.8% and the milk CF by 13.8%. The potential impact of bedding type on the CF reported herein is relevant and within the ranges observed by Aguirre-Villegas and Larson [36].

Manure is the second largest source of GHG emissions from dairy farms [36]. Therefore, manure handling strategies can be effective strategies to reduce non-enteric CH_4_ emissions and the milk CF. In the current study, the MED1 farm had a semi-solid manure collection system. The solid collection systems are the least contaminating in terms of GHG emissions compared with semi-solid, multiple and liquid systems [36,37]. Solid manure has less available water and is usually stored in stock piles that promote aeration, which reduces CH_4_ emissions. In contrast, liquid manure is stored on pits that promote anaerobic conditions, increasing CH_4_ emissions. Compared with slurry or liquid-slurry systems, MED1 farm has already reduced CH_4_ emissions and the CF by around 32 and 7%, respectively. However, moving MED1 semi-solid manure into a solid system would have an additional important impact on the CF (17.4% reduction). The installation of an anaerobic digestor could reduce enteric CH_4_ and the milk CF by 29.9 and 24.8%, respectively. Aguirre-Villegas and Larson [36] reported that anaerobic digestion can reduce GHG emissions related to manure management by more than 50%, mostly in the form of CH_4_ during storage. However, the largest contribution to the reduction of the milk CF would come from shifting an uncovered storage tank to an enclosed one that would reduce non-enteric CH_4_ and the CF of milk up to 35.7 and 27.5%, respectively. This large impact of an enclosed manure handling system agrees with Rotz et al. [13], who reported that enclosing manure storage with a flare to burn the escaping biogas would reduce by 39% the milk CF. Overall, manure handling has the largest potential to effectively contribute to the reduction of CH_4_ and the milk CF compared with those obtained through changes in dietary strategies. 

This study has several limitations. For example, the costs of the proposed mitigating strategies were not considered. Therefore, future studies aimed at estimating the cost of implementation of each strategy will help clarify the cost effectiveness of these mitigation strategies. The uncertainties and assumption in primary and secondary input data such as the average body weight, feed dry matter intake, ingredient and chemical composition of diets, fertilizers and manure application to croplands and pasture emissions factors may affect the accuracy of the results of this analysis using the IFSM. Rotz et al. [37] reported that uncertainties for IFSM predictions of GHG emissions were ±10 and ±20% for enteric and manure handling CH_4_, respectively, ±50% for N_2_O, and ±20% for fuel combustion and upstream emissions. In addition, the current analysis considered all relevant activities from cradle to farm gate, but excluded emissions attributable to imported feeds and its transportation. Finally, strategies to reduce CH_4_ emissions could be associated with other environmental impact such as water contamination, denitrification and eutrophication. Therefore, the milk CF analysis should be integrated with other footprint indicators (water, land and energy) in order to cover other environmental impacts spectrum of dairy farming.

## 5. Conclusions

The IFSM was used in this study to quantify the profile of GHG emissions in twelve dairy cattle farms from three Spanish regions. This study revealed that Cantabric farms are more efficient in term of GHG emissions compared with Mediterranean and Central dairy farms. CH_4_ was found to be the major contributor to the milk CF. Mitigation efforts are frequently targeted to changes in dietary strategies while management efforts are often ignored. However, simulation scenarios showed that CH_4_ emissions and the CF of milk could be reduced more through management practices compared with dietary strategies. Modelling may provide policy makers, farmers and stakeholders valuable information for planning and developing strategies to reduce the CF associated with milk production.

## Figures and Tables

**Table 1:** Characteristics of the selected dairy farms in Spain by region.

Parameters	Mediterranean Region Farms				Cantabric Region Farms				Central Region Farms			
	Mean	Max	Min	SD	Mean	Max	Min	SD	Mean	Max	Min	SD
**Farm structure**												
Farm size (ha)	154	330	25	127.7	56	82	25	26.5	105	180	60	52.3
Grazing area (ha)	-	-	-	-	14.5	40.0	0.0	19.00	-	-	-	-
Italian Ryegrass (ha)	13.5	26.0	0.0	11.70	14.8	26.0	4.0	12.42	-	-	-	-
Alfalfa (ha)	8.3	24.0	0.0	11.32	0.0	0.0	0.0	0.00	10.3	25.0	0.0	12.39
Maize (ha)	27.5	60.0	0.0	32.02	14.3	36.0	0.0	17.56	12.5	40.0	0.0	18.93
Sorghum (ha)	8.8	35.0	0.0	17.50	-	-	-	-	-	-	-	-
Oat (ha)	29.3	70.0	0.0	35.06	-	-	-	-	70.5	180.0	25.0	73.29
**Herd and production characteristics**												
Number of animals	280	440	106	154.9	161	240	64	72.6	319	400	198	88.3
Number of lactating cows	133	220	50	80.0	85	112	42	30.9	185	220	130	38.7
Number of dry cows	22	36	9	11.1	8	15	4	5.2	13	20	7	5.6
Heifers (1–2 years)	58	78	22	26.0	33	51	10	17.4	49	70	20	22.6
Calves	67	106	25	40.7	32	57	8	20.2	73	100	41	25.0
Milk yield (Tm/cow/year)	10.4	11.0	9.6	0.71	10.6	12.1	8.5	1.52	11.4	12.2	10.5	0.75
Milk protein (%)	3.6	3.9	3.4	0.23	3.4	3.8	3.1	0.30	3.3	3.5	3.2	0.15
Milk fat (%)	3.8	3.9	3.6	0.13	3.7	4.2	3.3	0.43	3.3	3.6	3.1	0.21
**Feed composition and intake**												
DMI ^1^ (kg/dairy cow/day)	24.5	25.8	22.3	1.53	23.5	25.2	21.3	1.64	25.6	27.2	23.8	1.73
% concentrates feeds	36	39	34	2.4	44	55	32	10.2	44	50	40	4.7
% forages	64	66	61	2.4	56	68	45	10.2	56	60	50	4.8
**Manure and fertilizer application**												
Liquid slurry application (m^3^/ha)	45	50	40	4.1	27	50	0	20.7	49	60	40	8.3
NPK ^2^ (kg/year)	264	300	230	30.4	219	300	0	146.4	194	275	80	82.2

^1^ DMI; dry matter intake; ^2^ NPK: nitrogen, phosphorus and potassium.

**Table 2:** The profile of greenhouse gas by source of emissions from the selected dairy farms by region.

Greenhouse Gas Emissions	Mediterranean Region Farms				Cantabric Region Farms				Central Region Farms			
	Mean	Max	Min	SD	Mean	Max	Min	SD	Mean	Max	Min	SD
**CH_4_ (kg/cow) ^1^**												
Animals and housing	202	217	180	17.2	110	194	25	93.3	187	205	168	17.0
Manure storage	126	152	96	23.8	56	91	14	40.3	86	166	33	60.8
Field-applied manure	0.2	0.4	0.1	0.14	0.1	0.2	0.0	0.08	0.3	0.6	0.1	0.24
Grazing	0.0	0.0	0.0	0.00	84.5	196.4	0.0	100.07	0.0	0.0	0.0	0.00
Total	328	352	292	25.5	251	284	180	48.0	273	335	223	52.0
**N_2_O (kg/cow) ^2^**												
Animals and housing	3.1	3.6	1.8	0.86	0.6	1.2	0.0	0.67	2.3	3.2	0.0	1.52
Manure storage	0.2	0.8	0.0	0.40	0.0	0.0	0.0	0.00	0.7	1.4	0.0	0.58
Cropland	3.2	4.8	2.4	1.13	2.7	5.7	1.0	2.17	1.0	1.6	0.6	0.43
Total	6.5	8.4	4.2	1.81	3.2	5.7	2.2	1.66	4.0	5.6	2.0	1.52
**CO_2_ (kg /cow) ^3^**												
Animals and housing	6391	6850	6107	340	3776	6721	751	334	6106	6474	5806	308
Manure storage	418	503	357	71.2	199	321	50	140.4	333	649	131	248.9
Net feed production	−10,553	−10,155	−10,776	275	−9551	−7431	−10,301	1414	−9681	−8843	−10,400	738
Fuel combustion	239	313	169	60.2	163	239	61	74.9	204	372	89	128.7
Grazing animals	0.0	0.0	0.0	0.00	2399	5541	0.0	2835.6	0.0	0.0	0.0	0.00
Net Emissions	−3506	−3075	−3909	465	−3014	−2516	−3398	367	−3038	−2787	−3162	173
**Total GHG (ton CO_2_-eq ^4^)**												
Animals and housing	670	1036	288	370.2	414	579	162	186.6	849	1045	536	218.8
Manure storage	554	841	182	338.6	161	310	19	134.4	1769	5143	348	2259.8
Net Feed production	122	204	36	68.8	67	172	30	70.0	78	184	5	78.5
Fuel combustion	38	49	7	20.4	15	25	2	9.4	48	75	26	19.9
Secondary sources	549	991	213	352.0	217	310	98	91.5	562	772	167	271.2
**CF (kg of CO_2_-eq/kg of ECM) ^5^**	1.0	1.1	0.8	0.12	0.7	0.8	0.6	0.07	0.8	0.9	0.8	0.08

^1^ CH_4_: methane; ^2^ N_2_O: nitrous oxide; ^3^ CO_2_: carbon dioxide; ^4^ CO_2_-eq: CO_2_ equivalent; ^5^ CF: carbon footprint expressed as kg of CO_2_ per kg of energy corrected milk with 3.5% fat and 3.1% protein concentrations.

**Table 3:** Enteric methane emissions evaluation from feed intake in the selected farms using the CNCPS ^1^ model.

Farm	Number of Milking Cows	Milk Yield (kg/day)	Enteric Methane (g/kg Milk)
Mediterranean Region (MED)			
MED1	82	32.0	12.3
MED2	59	36.5	11.2
MED3	180	34.5	13.1
MED4	220	33.0	13.3
Mean	135	34.0	12.5
Cantabric Region (CAN)			
CAN1	85	36.5	12.2
CAN2	42	28.5	16.2
CAN3	102	36.0	13.4
CAN4	112	40.0	12.4
Mean	85	35.3	13.6
Central Region (CEN)			
CEN1	220	36.5	14.4
CEN2	200	40.0	11.2
CEN3	190	34.0	13.2
CEN4	130	38.0	10.7
Mean	185	37.1	12.4

^1^ CNCPS: Cornell Net Carbohydrate and Protein System Model.

**Table 4:** Characteristics of the selected two extreme dairy farms in term of enteric and non-enteric methane emissions.

Parameters	Selected Dairy Farms	
	Farm MED1 ^1^	Farm CAN2 ^2^
Farm area (ha)	330	25
Soil type	Clay loam	Loam
Number of animals	197	64
Number of lactating cows	82	42
Number of dry cows	21	4
Heifers (1–2 years)	54	10
Calves	40	8
Calving strategy	Whole year	Whole year
Housing of animals	Free stall	Free stall
Milk yield (kg/cow/year)	9565	8500
Primary feed	Corn, sorghum and ryegrass silage; and concentrates	Pasture, ryegrass silage and concentrates
Grazing time	0	Whole day
Intake (kg DM ^3^/cow/day)	26.5	21.3
Type of manure	Semi-solid (12−14% DM)	Liquid-slurry (5−7% DM)
Type of storage pit	Top-loaded lined earthen basin	Covered basin
Storage period for manure	6 months	4 months
Bedding type	Straw (4 kg)	None
Enteric CH_4_^4^ emissions (g/kg milk)	12.3	16.2
Enteric CH_4_ emissions (kg/cow/year)	145	167
Non-enteric CH_4_ emissions (kg/cow/year)	191	29

^1^ Mediterranean region farm 1; ^2^ Cantabric region farm 2; ^3^ DM: dry matter; ^4^ CH_4_: methane.

**Table 5:** Potential of dietary strategies on the methane emissions change in the Cantabric region farm (CAN2).

Parameters	Baseline	Scenario 1	Scenario 2	Scenario 3
		Ratio F/C ^1^	Silage Quality ^2^	Ionophores ^3^
Dry matter intake (kg/day/cow)	21.6	21.6	21.6	21.6
Ration forage: concentrate	68/32	58/42	68/32	68/32
Milk yield kg/cow/day	28.5	31.2	28.7	28.6
CH_4_^4^(g/kg milk)	16.4	15.6	16.3	16.3
Total CH_4_ production (kg/cow/year)	170	177	170	170.4
% of CH_4_ change per kg of milk	0.0	−5.0	−0.5	−0.3
CF ^5^ (kg CO_2_-eq/kg ECM)	0.59	0.57	0.59	0.59
% CF change	-	3.5	0.0	0.0

^1^ F/C: 10% reduction in the forage/concentrate ratio; ^2^ Improved silage quality; ^3^ Supplement 330 mg monensin/day; ^4^ CH_4_: methane; ^5^ CF: carbon footprint.

**Table 6:** Potential of management practices change on methane emissions in the Mediterranean region farm (MED1).

Simulation Scenario	Baseline and Changes Details		CH_4_^2^ Emissions (kg/cow/year)	% CH_4_ Change	CF ^3^ (kg CO_2_-eq/kg ECM ^4^)	% CF Change
Change in milk production (kg/cow/year)	Baseline	9565	335.5	-	1.09	-
	Change 1	8000	335.1	+0.1	1.16	+6.4
	Change 2	11,000	334.8	−0.2	1.02	−6.4
Change in manure collection system ^1^	Baseline	Semi-solid	230.4	-	1.09	-
	Change 1	Solid	226.6	−1.6	0.90	−17.4
	Change 2	Slurry	335.1	+31.2	1.17	+7.4
	Change 3	Liquid-slurry	338.5	+31.9	1.17	+7.4
Change in manure storage method	Baseline	Top-loaded lined earthen basin	334.8	-	1.09	-
	Change 1	Top-loaded lined concrete tank	332.6	−0.7	1.09	0.0
	Change 2	Bottom-loaded tank or basin	301.9	−9.8	1.01	−7.3
	Change 3	Covered tank or basin	264.7	−20.9	0.93	−14.7
	Change 4	Enclosed tank	215.3	−35.7	0.79	−27.5
Change in bedding type	Baseline	Straw	335.5	-	1.09	-
	Change 1	Chopped straw	334.7	−0.2	1.09	0.0
	Change 2	Sawdust	334.8	−0.2	1.08	−1.0
	Change 3	Sand	302.2	−9.9	0.94	−13.8
	Change 4	Manure solids	302.5	−9.8	1.00	−8.3
Anaerobic digester installation	Baseline	No anaerobic digester	335.6	-	1.09	-
	Change 1	With anaerobic digestor	235.2	−29.9	0.82	−24.8

^1^ Manure collection system: Semi-solid (12–14% DM); Solid (20% DM); Slurry (8–10%DM); Liquid-slurry (5–7% DM); ^2^ CH_4_: methane; ^3^ CF: carbon footprint; ^4^ ECM: energy corrected milk with 3.5% fat and 3.1% protein concentrations.
